# “I can't stop worrying about everything”—Experiences of rural Bangladeshi women during the first postpartum months

**DOI:** 10.3402/qhw.v10.26226

**Published:** 2015-01-14

**Authors:** Maigun Edhborg, Hashima E Nasreen, Zarina Nahar Kabir

**Affiliations:** 1 Division of Nursing, Department of Neurobiology, Care Sciences and Society, Karolinska Institutet, Stockholm, Sweden; 2 Department of Community Medicine, Kulliyyah of Medicine, International Islamic University Malaysia, Kuala Lumpur, Malaysia

**Keywords:** Postpartum, depressive symptoms, content analysis, cultural traditions, Bangladesh

## Abstract

Over recent years, researchers have found evidence which indicates that the prevalence of postpartum depressive symptoms crosses cultural boundaries and is reported to be at least as high in non-Western countries as in Western countries. However, qualitative studies about new mothers’ experiences from non-Western countries, such as Bangladesh, are rare, particularly in rural areas. This study aims to describe the experiences and concerns of rural Bangladeshi mothers with postpartum depressive symptoms. Open narrative interviews were conducted with 21 mothers with depressive symptoms 2–3 months postpartum, consecutively selected from a longitudinal study about prevalence and risk factors of perinatal depressive symptoms. Inductive content analysis was used to analyse data and three themes emerged: family dynamics, living at the limits of survival, and role of the cultural context after childbirth. These themes were based on six categories and 15 subcategories. The findings show that troublesome family relationships, including intimate partner violence and violence in the family, influenced the mothers’ mental well-being. They and their families lived at the limit of survival and the mothers expressed fear and worries about their insecure situation regarding economic difficulties and health problems. They felt sorry for being unable to give their infants a good start in life and sad because they could not always follow the traditional norms related to childbirth. Thus, it is important to focus on the depressive symptoms among new mothers and offer counselling to those showing depressive symptoms, as the cultural traditions do not always alleviate these symptoms in the changing Bangladeshi society today.

Maternal depressive symptoms around childbirth have long been considered as culture-bound and rare or non-existing outside the Western cultures (Cox, 1996). Depressive symptoms in women after birth are supposedly related to the rise of the modern obstetric practice in the Western countries, which has alienated women from guidance and social support while they adapt to their new role as a mother (Hanlon, Whitley, Wondimagegn, Alem, & Prince, 2009). Thus, women from non-Western cultures have been thought to be protected from depressive symptoms by the rituals and restrictions that accompanied the transition to motherhood (Cox, 1996). However, over the recent years, researchers have found evidence that indicates that maternal postpartum depressive symptoms cross cultural boundaries, reporting the prevalence to be at least as high in non-Western countries as in Western countries (Affonso, De, Horowitz, & Mayberry, 2000; Oates et al., 2004).

In a systematic review by Gavin et al. (2005), the combined point prevalence of postpartum depression (PPD) was estimated to range from 6.5 to 12.9% during the pregnancy and the first-year postpartum, based on 28 studies from Western or high-income countries. The strongest predictors of maternal depressive symptoms postpartum have been reported to be a past history of psychological disturbances during pregnancy, poor marital relationship, low social support, and stressful life events (O'Hara & Swain, 1996). In a recent systematic review from low- and middle-income countries (including Bangladesh), Fischer et al. (2011) cited a prevalence of almost 20% for common mental illnesses, such as depression and anxiety postpartum. The strongest risk factors reported by Fischer et al. (2011) were, for example, socio-economic disadvantages, a lack of intimate empathy, a lack of emotional and practical support, having hostile in-laws, and having an experience of intimate partner violence. In a longitudinal study among rural Bangladeshi women, the prevalence of depressive symptoms was found to be18.3% during pregnancy, 14.1% at 2–3 months, and 31.7% 6–8 months postpartum (Nasreen, 2011).

In high-income countries, childbirth is rarely a life-threatening event any longer (Eberhard-Gran, Garthus-Niegel, Garthus-Niegel, & Eskild, 2010). A vast majority of women give birth in hospital, and the mother and infant are usually discharged from hospital within 2 days after giving birth after being provided with instructions concerning infant feeding, diet, and exercise (Jain & Levy, 2013).

Compared to the Western countries, giving birth in Bangladesh is still hazardous for women and 76% of the deliveries take place at home with help from traditional birth attendants. Almost 80% of maternal deaths occur in the rural parts of the country (Mamun et al., 2012). In Bangladesh, a traditional practice is for the new mothers to rest for 40 days after childbirth, to eat a special diet, and stay indoors during the first postpartum period. As the new mother is regarded “unclean” due to menstrual bleeding, she is not allowed to either wash or prepare food, and therefore housework is generally done by the others (Eberhard-Gran et al., 2010).

In a literature review of 14 studies from five continents, Bina (2008) focused on the association between cultural factors and postpartum depressive symptoms and arrived at some conflicting results. In eight of the studies, she found that cultural rituals had an alleviating impact on the depressive symptoms and that a lack of cultural traditions led to increased prevalence of depressive symptoms postpartum. One study reported a lack of association, and the other suggested that cultural traditions could contribute to postpartum depression. Bina (2008) emphasised the importance of the mothers’ perception of support as being satisfactory. In some cases, women did not perceive the traditions as being supportive, for example, when they experienced difficult relationships with those providing the support. Oates et al. (2004) found in their cross-cultural study about maternal depressive symptoms postpartum in 11 countries from five continents, that the mother-in-law could be a source of unhappiness for new mothers following childbirth in all involved countries, except Sweden.

According to Klanin and Arthur (2007), sex preference in favour of sons is deeply ingrained in Asian countries. Many researchers from Asian countries have reported a direct association between an infant's female sex and postpartum depression (Chandran, Tharyan, Muliyil & Abraham, 2002; Patel, Rodrigues, & de Souza, 2002), which includes Bangladesh (Gausia, Fisher, Ali, & Oosthuizen, 2009).

Qualitative studies about women's own experiences of depressive symptoms postpartum are rare in low- and middle-income countries. In a phenomenological study, Gao, Chan, You, and Li (2009) found that the depressed mothers in China felt physically and emotionally exhausted, they perceived themselves as incompetent mothers, and they experienced dissonance between tradition and modernity and between expectations and reality. The practice “of doing the month”—postpartum rules which women comply with to restore balance after childbirth (Eberhard-Gran et al., 2010), the daughter-in-law/mother-in-law relationship, the sex of the baby, and the one-child policy contributed to their depression, according to the mothers (Gao et al., 2009). In a qualitative study in India, mothers reported that the most common explanation for the mother's feelings of depression was economic causes and a poor relationship with family members, particularly the husband and mother-in-law (Rodrigues, Patel, Jaswal, & de Souza, 2003). In Ethiopia, new mothers with postpartum mental distress experienced disappointment and exclusion, an exacerbation of pre-existing problems, and felt vulnerable and exposed to danger (Hanlon et al., 2009).

Due to the high prevalence of depressive symptoms in rural Bangladeshi women (Gausia et al., 2009; Nasreen, Kabir Nahar, Forsell, & Edhborg, 2011) and low numbers of qualitative studies from South Asia, particularly from rural Bangladesh, about new mothers’ experiences of postpartum mental health, further research is needed to get a better understanding of the kind of support and health services that should be developed to address the challenge. In this study, we aimed to explore and describe the experiences and concerns during the first 3–9 months following childbirth of those mothers who showed depressive symptoms 2–3 months postpartum, in a rural area in Bangladesh.

## Methods

### Study setting and design

This qualitative study is part of a longitudinal, cohort study “Risk factors and consequences of maternal perinatal depressive and anxiety symptoms: A community-based study in rural Bangladesh” (Nasreen, 2010), which was carried out in two subdistricts of Mymensingh district, 120 km north of the capital Dhaka in Bangladesh. The district is a rural area with a population of 4.1 million, characterized by homogeneity in terms of ethnicity, culture, and language (UNICEF, 2008).

Ethical approval for the study was received from The Bangladesh Medical Research Council (Ref. no. BMRC/Eth.C/2008/402) in Bangladesh and the Regional Ethical Review Board at Karolinska Institutet, Stockholm, Sweden (Ref. no. 2008/919-31).

### Participants and procedures

Participants for the study were selected from a sample of mothers identified as having depressive symptoms 2–3 months postpartum in the longitudinal study, that is, having a score of 10 or more on the Edinburgh Postnatal Depressive Scale (EPDS; Cox, Holden, & Sagovsky, 1987). The mothers were approached consecutively by a trained female Bangladeshi anthropologist, informed about the study, and asked if they wanted to participate in an open narrative interview. Twenty-one mothers of infants (both primi- and multiparas)— whose infants were between 3 and 9 months old (*M*=6.2 months) were approached, informed, and agreed to participate.

The open narrative interviews of approximately 2 h were conducted between January and July 2009 in Bangla by the trained Bangladeshi anthropologist at the women's homes. Except for some background data, the interviews were loosely structured and the interviewer asked the woman to narrate her experiences and concerns after childbirth. If the mother had difficulties narrating freely, then she was asked specifically about the delivery; the postpartum period; social support; breastfeeding; relationship to the infant, partner, and family; and health in the family. All interviews were tape-recorded and transcribed verbatim in Bangla and translated into English prior to coding. One of the co-authors (ZNK) who is bilingual read all the interviews in Bangla and controlled the English translation against the Bangla version. In case of a discrepancy in the translation between Bangla and English, it was discussed between the two bilingual authors (ZKN and HEN).

### Data analysis

The data were analysed using inductive content analysis (Granehielm & Lundman, 2004). Content analysis is a research technique for making valid inferences from texts to the contexts of their use (Krippendorff, 2013).

The first author (ME) read through the English translations of the interviews several times to obtain a sense of the whole. One bilingual researcher (ZKN) read all the interviews in Bangla and another (HEN) read half of the interviews. For each transcript, the first author (ME) identified the meaning units related to the aim, marked them, and condensed the meaning units. The condensed meaning units were then abstracted and labelled with a code according to Granehielm and Lundman (2004).

To increase the credibility of the analysis, a bilingual anthropologist and the first author (ME) independently coded four interviews in English. The coding schemes for these four interviews were compared and found mostly to be in agreement. The first author (ME) then coded the remaining transcripts, drawing upon additional codes where required from the data. The various codes were then compared based on differences and similarities and sorted into subcategories and categories, that is, the manifest content (Granehielm & Lundman, 2004). Subsequently, the tentative categories and subcategories were compared and discussed between the three authors until agreement was reached. Finally, the underlying meaning, that is, the latent content of the categories (Granehielm & Lundman, 2004), was formulated into three themes.

## Results

The narrations of the new mothers about their experiences and concerns after birth related to three themes: 1) family dynamics; 2) living at the limits of survival; and 3) role of the cultural context after childbirth. We present the findings according to these three themes, six categories and 15 subcategories (Table I).

**Table I:** Themes, categories, and subcategories emerging from the women's narratives

Themes	Categories	Subcategories
Family dynamics	Family relations	Living together with in-laws Polygamous marriages
	Violence	Intimate partner violence Violence in the family
Limits of survival	Poverty	Economic difficulties Not enough food
	Insecurity	Fear of dying during the delivery Earlier negative life events
	Health	Maternal physical health Maternal mental health Infant health
Role of the cultural context	Traditional norms	Birth in the father's house Restrictions Support from own family Preferences for boys

Twenty-one women, all of them showing depressive symptoms at 2–3 months postpartum (*M*_EPDS_=11.8, EPDS range: 10–14), and most of them (except two) also during the third trimester of the pregnancy (*M*_EPDS_=11.4, EPDS range: 6–29), were interviewed (Table II).

**Table II:** Background data of the participants

	Participants *N*=21
Mother's age [Md (min–max)]	27 (17–45)
Husband's age [Md (min–max)]	36 (25–55)
Mothers literate [*N* (%)]	
Yes	11 (52%)
No	10 (48%)
Parity [Md (min–max)]	4 (1–7)
Previous loss of child [*N* (%)]	
Yes	9 (42%)
No	12 (48%)
Delivery [*N* (%)]	
Home	19 (90%)
Hospital	2 (10%)
Sex of the infant [*N* (%)]	
Boy	11 (52%)
Girl	10 (48%)
Age of the infant/months [Md (min–max)]	6 (3–9)
Mothers’ EPDS during pregnancy [Md (min–max)]	12 (6–29)
Mothers’ EPDS at 2–3 months [Md (min–max)]	11 (10–14)
Mothers’ EPDS at 6–8 months [Md (min–max)]	12 (11–22)

### Family dynamics

#### Family relationship

All marriages, except one, were arranged by the parents or family. Several of the mothers were married to men who had been married before and were thus responsible for step-children after the marriage. Most of the mothers lived together with their in-laws in the same bari (several households sharing same courtyard) (Kabir, 2001), but most of them had separate households. In a few cases, the mothers lived in polygamous marriage.

Although most women reported supportive relationships with their husbands and in-laws, they admitted that they also had fights and quarrels in the family: “There is always a quarrel in a family when money is needed” (*32 years, 3-para*). Those women who were living in polygamous marriages, that is, in the same household with the husband's other wife said that they always quarrelled, mostly about the children and particularly if the children suffered from illness and needed medicine. The mothers reported having a good relationship with their infant. They said they loved the baby and were happy irrespective of whether they had a son or a daughter. However, some mothers reported that their babies had sleeping problems and that the baby had been crying at night for 2–3 months. Other mothers reported eating problems and these mothers were very concerned about their infants’ low weight.

#### Violence

The women reported on intimate partner violence and that they were sometimes beaten by their husbands. However, in most of these cases they did not feel so bad about it: “He becomes angry and beats me, but it is no big deal!” (*30 years, 7-para*). “He could slap me, but then he would go out and calm down” (*27 years, 2-para*). However, the intimate partner violence could be worse and one woman, whose husband already had one wife, said: “He just beats me … ever since this woman (the first wife returned after husband's second marriage) came, he can't stand the sight of me” (*22 years, 4-para*).

Even if there was no intimate partner violence, several women reported problematic relationship and sometimes violence in the family and that they were scared of other family members. For one woman, the violence from in-laws during the pregnancy was so bad that it was reported to the police. This mother said: “I am scared of them (the parents-in-law), they look at me threateningly, and they want to pick issues. When they come home, I stay in my room and don't go out” (*20 years, 2-para*).

The mothers also reported conflicts about inheritance in the families. In one family, the mother said that there was quarrelling and fights between her and her brother-in-law after her father-in-law's death because she thought her “brother-in-law will swindle her husband … because he is not too sharp” (*24 years, 3-para*). Another mother left her husband before the birth of her daughter, due to violence from both her husband and in-laws during her pregnancy because they wanted more money as a dowry.

### Limits of survival

#### Poverty

All the women reported that their families were poor and had a difficult economic situation, except for two women who said they “had enough to eat, but not to sell” (*45 years, 5-para*) and “not too much and not too little” (*36 years, 7-para*). These families had their own land for cultivation. Mostly, the women's husbands did farming on others’ lands and several families did not have any steady income due to the husbands’ unemployment, irregular employment, or poor health, which made the husbands unable to work full time. The mothers spoke a lot about financial worries and that the families “suffer hardships” and “live hand to mouth” (*29 years, 6-para*).

The majority of the women expressed that they did not have enough to eat. With a single income, which most of the families lived on, the mothers had difficulties in properly feeding all: “It is difficult to depend on one person only in such a big family” (*36 years, 7-para*). Another mother said, “I have a big sorrow in my heart … I cannot look after my baby the way I would like to. I can't feed him properly. I have such pain in my heart for this” (*20 years, 2-para*).

#### Insecurity

Pregnancy and childbirth were very insecure periods for these poor women and were associated with risk for the woman's life. Several mothers said that they had fear of dying during the delivery, and expressed fear for what would happen with the children if they died: “Who will look after them, love them if my husband marries again?” (*36 years, 7-para*).

The women expressed fear that complications during the pregnancy may force them to go to the hospital for the delivery and put a strain on their economic situation. Only two women had their children at hospital, although several had complicated pregnancies with lots of problems and pain. Particularly those women who had many children expressed that they were very weak already before the delivery and that they experienced many difficulties during labour due to their poor health. The women were always afraid and worried about unpredictable events, such as illness in the family and needing doctors and medicine, because they did not have any money saved for unpredictable events. The women whose husbands had more than one wife felt insecure in their marriages, as well as those mothers who did not have any sons. Another source of insecurity was that their husbands did not want birth control and one mother was afraid that she was already pregnant again.

One kind of insecurity consists of earlier negative life events, which still influenced the daily lives. Approximately one-third of the mothers had lost at least one child previously, many of them during childbirth, and these mothers experienced pregnancy and labour as very stressful and frightening. Some women reported that their husbands had accidents (traffic- and work-related) several years ago, which still affected their lives through the husbands’ decreased ability to work full time, needing expensive medicine, and visits to the hospital and doctors. Those women who had lost their parents during childhood felt insecure as they did not have parents to look after them. The parental family seemed to be a secure base for the women, whom they could call upon or go to when things became problematic in their new families.

#### Health

The mothers with depressive symptoms reported poor physical health after birth. They felt weak and had no physical strength which they explained through loss of blood during delivery or as lacking calcium and vitamins. They complained about multiple health problems, such as stomach pains, chest pains, headache, dizziness, low or high blood pressure, jaundice, urinary problems, gastric problems, a tumour in the throat, and having trouble walking after childbirth. The mothers reported that they had lost weight, became thin, and lacked an appetite, and they related their health problems to worries for the children, for financial issues, and for not being able to give the children enough food or to raise and bring them up properly.

About their own mental health, the mothers talked about worries and tension in their lives and that “things are running in their heads” and they “can't stop worrying about everything” (*30 years, 7-para*). Some mothers said they had problems concentrating, they can't remember anything, and they had problems working properly and felt mentally weak. One mother said: “I feel sad in my heart” (*17 years, 1-para*) and another said: “I have mental anxiety and depression. My husband has another wife, they show love for each other which makes me unhappy” (*32 years, 3-para*).

The majority of the mothers were worried and anxious for their infants’ health. The infants had been ill several times with fever, cough, measles, and dysentery. Several mothers reported that their infants have had pneumonia. The majority of the mothers expressed that their infants were very thin, had lost weight, and were less active, particularly after the breastfeeding stopped. One mother said, “I just look at how thin my baby is. I don't know what I am supposed to give her” (*25 years, 3-para*). One infant was described by his mother as blind and not interested in other people. It was the mother's first child after a very complicated delivery and she felt very sad: “I am more worried now about the baby …. If my baby stays healthy, I will be in peace” (*17 years, 1-para*).

### Role of the cultural context

#### Traditional rules

The women were well aware of the traditional customs around childbirth, but only a few mothers followed the custom of “giving birth in their father's house” to get support from her mother and female relatives. A majority of the women wanted to go to their parental home, but some of them preferred to give birth at their marital home, particularly if their biological mother was deceased. As reasons for not going to their parental homes, the women expressed that their husbands and in-laws did not allow them to go or because they did not want to leave for practical reasons, such as leaving the house empty, that the husbands had to work for an income and could not do all the household work himself or, as one woman said, he was “tired of cooking for himself” (*27 years, 2-para*).

Traditional food restrictions were followed by a majority of the women but not all. One mother said: “They now say that you lose breast milk if you follow the diet, if I have fish and milk, then there will be plenty of breast milk” (*20 years, 2-para*). Another mother said: “These rules, you know, we do not actually follow them anymore … but it is better to follow them, the baby stays healthier this way” (*28 years, 5-para*). The mothers believed in the “evil eye” and that both they themselves and the baby could be jinxed. They said they were scared to go out in the evenings, fearing that something might happen to the infant.

The *restriction* of staying indoors was followed in various ways. Very few stayed indoors for 40 days. Most said that they stayed indoors for 14–15 days. In contrast, all new mothers, also those who did not follow the diet or indoor restrictions, adhered to the practice of not having sex with their husbands for 40 days after childbirth. However, several of the women could not follow the rule because their husbands insisted on intercourse. “I had to be with him. I can't say no to my husband, it is a sin to deny him” (*30 years, 7-para*).

Those mothers who delivered their child at their parental house were satisfied with the support from their own families, particularly from their mothers and sisters. Even if they gave birth in their marital house, their own mothers and sisters seemed to be the greatest source of support, and also the mother-in-law and elder children helped. Husbands often did the heavier work and took care of the other children in the family. One mother said: “Everybody has to do their part” (*24 years, 3-para*).

A few mothers reported that they did not cook for 40 days, but most of them said that they started to do easy household work after 14 days, sometimes even after 7 days because there was no option. Some mothers said that their husbands did not help in the housework and one mother said: “My husband does not do women's work” (*41 years, 7-para*). These mothers felt helpless and bad for not having enough help, particularly when they had to start cooking for their family only 14–21 days after childbirth.

Although the mothers often said that they did not have any preferences for the sex of the child, many of the mothers said that they wanted sons and one mother said: “A boy means peace in later life” (*25 years, 3-para*), and if these mothers who wanted a boy instead had a girl, they admitted that they felt sad for having a girl. “Not disappointed. But I feel a little bad. Everybody wants a boy” (*20 years, 2-para*). The mothers said that neighbours and relatives gossiped and criticized the mother if there were several girls in the family and they admitted that it hurt. “I feel a bit hurt when I hear their words” (*30 years, 7-para*). The mothers did not feel criticized by their husbands, but in one family the husband talked about marrying again. This mother said: “If I had had a son this time, I wouldn't have any more sorrows” (*27 years, 2-para*). However, if the mothers had sons earlier, they often wanted a girl because as one mother said, “you need both. A son alone cannot bring happiness, neither can a daughter” (*36 years, 7-para*).

## Discussion

### Worries related to poverty, insecurity in life, and health

The mother's narratives included a lot of worries, anxiety and fear, mostly related to poverty and insecurity in their lives. Similar to Hanlon et al. (2009), we found that pregnancy and childbirth seemed to exacerbate pre-existing problems in the family. The birth of a child meant one more to feed and increased risk for the mother and baby to become ill and thus, for unexpected expenses for medicine, medical consultations, and hospital stays. The mother's confinement after birth required more support from her spouse and family, which was not always accessible. The worries were seen by the mothers as the reason for feeling mentally weak. They also explained their poor physical health, thinness, and lack of energy and strength as a result of their many worries.

Fischer, Tran, and Tran (2007) did not find any associations between psychosocial risk factors such as a lack of support, negative life events, and postnatal depressive symptoms in their study from Vietnam, which are usually reported in high-income countries as risk factors for maternal depressive symptoms postpartum (Cooper & Murray, 1998). Instead, the risk factors in Vietnam were socio-economic, such as a lack of salaried work or an insecure source of income (Fischer et al., 2007). This risk factor was very apparent among our participants, where the mothers described having many worries about poverty and insecurity in life due to their powerless situation which depended on their husband's discretion.

Mothers in our study, who complained of physical and mental ill health after birth, also reported having negative experiences of pregnancy and child delivery. This finding was confirmed in a recently published quantitative study by Gausia et al. (2012), who showed that postpartum depressive symptoms in Bangladesh was significantly associated with women reporting negative childbirth experiences. In a cross-cultural study, Oates et al. (2004) also reported physical, traumatic deliveries as a contributor to unhappiness. This indicates the need for skilled help during delivery to prevent birth complications, for example, trained midwives to help with the delivery in the women's house and support for those new mothers who reported negative birth experiences, because they are more likely to develop depressive symptoms.

Beck (2002) showed in a meta-synthesis of 18 qualitative studies of postpartum depression that in high-income countries, mothers who experience depressive symptoms after childbirth often defined themselves as bad mothers and reported pervasive feelings of loss of self. This was not so clear among the mothers in our study. Consistent with findings by Abrams and Curran (2011), mothers in the current study often attributed their worries to poverty and expressed feelings of not being able to provide their children with nutritious food or take care of them properly. Thus, they often referred to social reasons for their mental ill health, which were beyond individual control, as also reported by Fischer et al. (2011). This is a logical reaction to the mothers’ vulnerability around childbirth. These mothers need help from the society to get a less troublesome life situation, as well as support from ante- and postnatal care services. They need help to recover from both their own physical weakness and their psychological and emotional ill health; and support and education about their infants’ health.

### Sorrows related to the relationship with husband, family, and infant

In a literature review, Fischer et al. (2011) reported that except for socio-economic circumstances, the quality of the relationship with an intimate partner which includes violence and polygamous marriages, poor family and social relationships, and a lack of social support, were determinants of anxiety and depressive symptoms. Similarly, in the longitudinal study from which the current sample was selected, we found that poor relationships with a partner and physical intimate partner violence were significant predictors for maternal depressive symptoms 6–8 months postpartum (Kabir, Nasreen, & Edhborg, 2014). These were confirmed by the mothers’ own words in the current study. Although they talked a lot about worries and tensions related to poverty and financial insecurity in their living situations, they talked about sadness and sorrows in their hearts related to their relationship with the husband, the infant, and the family. One mother felt sad because her husband had another wife, whom her husband showed feelings for. Another felt sad because her husband cannot stand her since his first wife came back into their lives. These mothers clearly described their status of being one of two wives as the reason for their sadness. Polygamous marriage is reported as a risk factor for postpartum depressive symptoms in Nepal (Ho-Yen, Tschudi Bondevik, Eberhard-Gran, & Bjorvatn, 2007) and in Nigeria (Fatoye, Adeyemi, & Oladimeji, 2004).

### The role of the cultural traditions

Bina (2008) reported that cultural factors could be alleviating, deteriorating, or neutral in relation to postpartum depressive symptoms. The mothers in our study described that the cultural traditions could be alleviating and helpful when they could follow them. Only a few of the mothers went to their parental house for childbirth, and those mothers who did not expressed that it was difficult for them to get help for 40 days as per tradition. They often had to start household work 14–15 days after childbirth or even earlier. If these mothers wanted to follow the traditional rules but it was not possible due to practical reasons, the cultural factors could be experienced as disturbing, and made the mothers feel bad and afraid that negative incidents would happen to herself or the baby. A similar finding was reported by Fischer et al. (2007) about new mothers in Vietnam. Those who were given less than 30 days of rest after childbirth were at an increased risk for common perinatal mental disorders. However, not all mothers wanted to follow the traditional rules, particularly the diet and indoors restrictions. They often referred to doctors, mass media, or more educated relatives and said, for example, that if they ate meat and fish they would get plenty of breast milk.

Dressler, Baliero, Ribeiro, and dos Santos (2007) have developed a theory to examine how culture, conceptualized as a property of social groups, translates into effects on individuals. They described the concept cultural consonance as “the degree of which individuals, in their own beliefs and behaviours approximate the prototypes for belief and behaviour encoded in shared cultural models” (pp. 2058–2059). High cultural consonance has been associated with less psychological distress. Low cultural consonance in cultural domains with high cultural consensus or sharing might lead to depressive symptoms. In our study, the cultural consonance was high, and only a minority of women did not believe in the cultural traditions and rules. Thus, the cultural traditions seems to be protective for the women who could follow them, but for the many women who found it impossible to follow them, due to practical reasons, it could result in low cultural consonance and could therefore increase their psychological distress and anxiety and trigger depressive symptoms.

As others (Fischer et al., 2011; Klanin & Arthur, 2007) have reported, some mothers in this study expressed concerns that giving birth to a daughter rather than a son is a threat to her marital relationship. Consistent with other research (Leung, Arthur, & Martinson, 2005; Rahman, Iqbal, & Harrington, 2003), the new mothers in our study expressed that they felt blamed and criticized for having a girl, particularly if they already had several girls. These mothers felt very insecure and were afraid of being left by their husbands. Thus, although the women themselves did not express any preference for sex of the child, the preferences for boys in the society gave negative consequences for the individual families in case of having a girl child.

### Limitations of the present study

Although we tried to control for error in the translation between Bangla and English, some misinterpretations might have taken place. All women had relatively low EPDS scores in our study, indicating low depressive symptoms. It may also be indicative that using an instrument developed in the Western world was not appropriate in identifying the most depressed women. However, EPDS has been validated in Bangladesh and we followed the cut off with the highest sensitivity and specificity (Gausia, Fisher, Algin, & Oosthuizen, 2007). Another explanation could be that the women's circumstances in this study might result in more anxiety, worries, and fear than depressive symptoms.

## Conclusions

Although the women in this study showed depressive symptoms as assessed by EPDS, they talked more about worries and anxiety than sadness and unhappiness. This may be explained by the women's insecure and vulnerable situation due to financial constraints, poor physical and mental health, and their experience of domestic violence. It indicates a need for protection by the community as they did not always trust their husbands or did not have their own parents for support. Our findings suggest that accessibility to trained midwives may alleviate insecurity and fear during the delivery. After childbirth, it is important to pay attention to anxiety and depressive symptoms among the new mothers. Those showing anxiety or depressive symptoms can be helped with counselling, as due to practical constraints, the cultural traditions do not necessarily alleviate depressive symptoms in the changing Bangladeshi society today.
